# Evaluation of the effects of aerobic training during hemodialysis on autonomic heart rate modulation in patients with chronic renal disease

**DOI:** 10.1097/MD.0000000000015976

**Published:** 2019-06-07

**Authors:** Mauro J.D. Morais, Rodrigo D. Raimundo, Fabiano S. Oliveira, Luiz C. de Abreu, Italla M.P. Bezerra, Romeu P.M. Silva, Alliny S. Rodrigues, Vitor E. Valenti, Andrés R. Pérez-Riera

**Affiliations:** a Federal University of Acre, UFAC, Acre; b Graduate, Research and Innovation Sector, Santo Andre, São Paulo, ABC Medical School, FMABC; c Laboratory of Study Design and Scientific Writing, ABC Medical School, Santo André, Sao Paulo; d Postgraduate Program in Public Policies and Local Development, School of Sciences of the Santa Casa de Misericórdia de Vitória, EMESCAM, Vitória, Espirito Santo; e Paulista State University (UNESP), Center for the Study of the Autonomic Nervous System, Marília, Sao Paulo, Brazil.

**Keywords:** aerobic exercise, autonomic nervous system, chronic renal disease, hemodialysis, heart rate variability

## Abstract

**Introduction:**

Chronic renal disease (CRD) affects a large portion of the population and is directly related to cardiovascular problems and hypertension, among others. Studies show that heart rate variability is directly affected by these problems. Physical-oriented exercises have been shown to be of fundamental importance in improving the adverse effects to dialysis treatment.

**Objective:**

To analyze the effects of aerobic training during hemodialysis on autonomic heart rate modulation in patients with CRD.

**Method:**

Experimental study of an open, single group clinical trial. In this clinical trial, patients with CRD will initially undergo international physical activity questionnaire and kidney disease quality of life short form protocols, as well as monitoring of heart rate systolic, and diastolic blood pressure. After evaluation of the initial parameters, patients will undergo an aerobic exercise program for 12 weeks, in 3 weekly sessions, lasting 30 minutes a session. These evaluations will allow for a greater control of the disease, and monitoring of any improvements in the quality of life and self-esteem of these patients.

**Ethics and dissemination:**

This study was approved following the guidelines and norms that regulate research involving human subjects, in Resolution No. 466/12 of the National Health Council. It was approved by the Research Ethics Committee of the Faculty of Juazeiro do Norte, with the number 1962 092. All patients who agree to participate in the research will sign the informed consent form. The results will be disseminated through peer-reviewed journal articles and conferences.

## 1 Introduction

The World Health Organization (WHO) reports millions of deaths each year from chronic diseases. Among these chronic diseases, chronic renal disease (CRD) is considered a worldwide public health problem due to the high mortality rates.^[1]^ CRD has received increasing attention from the international scientific community, as its high prevalence has been demonstrated in recent studies. In Brazil, comprehensive epidemiologic studies on CRD using the new definition of the disease have not yet been performed.^[2]^ While the number of Brazilians in the different predialysis stages of CKD (Chronic renal kidney) is not known accurately, an analysis of adult laboratory data using the new definition of CKD revealed that 2.3% of subjects had glomerular filtration rate (GFR) <45 mL/min/1.73 m^2^ or chronic renal disease (CRD) stages 3B, 4, and 5. Extrapolating these results to the Brazilian adult population, it is suggested that about 2.9 million Brazilians would have a 3rd or less of the GFR of normal individuals.

The CRD is the slow, progressive, and irreversible loss of renal function causing the body to no longer be able to maintain metabolic and hydroelectrolyte balance.^[3,4]^ It causes serious consequences for the patient and his/her family, compromising physical, psychic, and social health, and will eventually directly affect their quality of life (QoL).^[3,4]^

Patients with CRD have shown an increase in survival due to the use of renal replacement therapy. The renal therapy of choice is a successful renal transplant, but hemodialysis and peritoneal dialysis present similar clinical results, and so are the most commonly used treatment.^[3,4]^ Patients with CRD are using hemodialysis and dialysis therapies, and they are likely to have sequelae in all areas of their life.^[3,4]^

The population of patients with CRD on hemodialysis is associated with several factors, such as: high morbidity rate, mortality, low cardiopulmonary capacity, low functional capacity, cardiovascular diseases, and arterial hypertension.^[5,6]^ Patients on chronic hemodialysis with cardiovascular disease frequently die suddenly, resulting in a high mortality rate. Heart rate variability (HRV), which describes oscillations in the intervals between consecutive heart beats (RR intervals), is influenced by the activity of the autonomic nervous system (ANS) on the sinus node. Reductions in HRV are prognostic of an increased risk of death in several populations, but its prognostic value in patients undergoing hemodialysis remains unclear.^[7]^ In addition, there is a very close relationship between CRD and systemic arterial hypertension, which could affect the cardiovascular system, and result in the dysfunction of autonomic cardiac modulation, with predominant sympathetic autonomic modulation. ANS dysfunction is commonly present in patients with CRD and patients undergoing hemodialysis, and is an independent factor for a poor prognosis and sudden death in this population.^[7,8]^ Cardiac autonomic dysregulation, shown by a reduced HRV, contributes to the high mortality observed in patients with CRD undergoing hemodialysis.^[9]^

The HRV is a noninvasive technique whose analysis can be performed using linear time and frequency and nonlinear methods in the chaos domain.^[10]^ Time domain methods use mathematically simple techniques to measure the variability present in the RR intervals by calculating the mean and standard deviation of heart rate variations over time, while frequency domain methods use the spectral analysis that monitors the decomposition of heart rate variation at a given time in its fundamental oscillatory components, that is, the time series is decomposed into different frequency components.^[10,11]^ Among the methods used for HRV analysis are the geometric methods, triangular index, triangular interpolation of the RR intervals (TINN), and Poincaré plot, which present the RR intervals in geometric patterns and use approximations to derive the measures of HRV.^[10,11]^

Knowledge of the physiologic responses involved in the exposure of patients with CRD to hemodialysis is important for the development of future therapies to monitor, intervene, or even prevent the development of cardiovascular system disorders.^[2]^ The important reduction in the functional capacity of patients with CRD treated with hemodialysis has been partially reversed with regular aerobic physical exercises.^[12]^ In addition, it may also contribute to the reduction of osteomyoarticular problems, improvement of the QoL, and improved adherence to the treatment plan.^[13]^ The National Kidney Foundation recommends that regular physical activity be part of the strategic management of patients with CRD.^[14]^

Physical training in normal individuals results in an increase in functional capacity, reduces the risk of cardiovascular diseases, and improves the psychologic structure.^[14]^ A similar benefit has been reported with dialysis patients, even though the actual impact is impaired by the difficulty of adherence to any exercise program, both in diseased and healthy individuals.^[15,16]^ Several studies have reported the effects of aerobic training in dialysis patients. The type and duration of the training programs are variable (8 weeks to 6 months), but all studies compared the patients before and after the training program.^[15,16]^

Patients with CRD on hemodialysis generally have a poor physical and psychologic condition, but we intend to demonstrate that a program of aerobic physical exercise, when well prescribed and monitored, is safe and can generate benefits for the treatment of these patients.

In this way, the present study has as general objective to evaluate the effects of aerobic training during hemodialysis on autonomic heart rate modulation and quality of life in patients with CRD, and objective specifics are as follows:

1. Describe the linear and nonlinear indexes of HRV in patients with CRD on hemodialysis during supervised aerobic exercise in cycloergometry.
2. To evaluate the quality of life in patients with CRD on hemodialysis during supervised aerobic exercise in cycloergometry.

## 2 Methods

This study protocol will follow the items of the Standard Protocol for Randomized Trials (SPIRIT). This is an open, single-group experimental study on the autonomic heart rate modulation response under the effects of aerobic training during hemodialysis sessions in patients with CRD.

### 2.1 Study population

Patients with CRD, who are on hemodialysis, at the Hospital das Clinicas in Rio Branco, Acre State, Brazil, will be included if they meet the inclusion/exclusion criteria described in the following section.

### 2.2 Inclusion and exclusion criteria

Adult patients of both sexes who have not been physically active for at least 6 months will be included. The patients have been under treatment for at least 6 months and have been clinically authorized by the physician responsible for the sector to be included in a physically active study.

Patients with diabetes mellitus, unstable angina, uncontrolled arterial hypertension (systolic blood pressure [BP]: 200 mm Hg and/or diastolic BP: 100 mm Hg), use antiarrhythmic drugs, severe pneumopathies, acute systemic infection, renal osteodystrophy, and severe, disabling neurologic, and musculoskeletal disorders will be excluded from the study.

### 2.3 Expected risks

The risks are minimal, but patients may feel constrained by the exercise sessions proposed in this work and so may not wish to participate in the research. Patients may also experience dizziness, nausea, cramps, fatigue beyond the supported, and headaches. In cases of clinical problems, activities will be discontinued.

### 2.4 Expected benefits

Relevant scientific information will be obtained on the recovery and rehabilitation of patients with CRD through the analysis of HRV after aerobic exercises using the cycloergometer. This will demonstrate that well-controlled aerobic physical activity can contribute to a better QoL and the psychosocial well-being of these patients, thus helping to prolong their life.

### 2.5 Intervention

Liao et al^[17]^ used 2 groups of 20 patients each, performing a 30-minute exercise program consisting of a 5-minute warm-up, 20-minute electric bicycle, and a 5-minute cooling down regimen during treatment with hemodialysis in the renal unit. The exercise was performed under the supervision of a physician and a rehabilitation nurse. This was adapted in this study to be conducted in a single screening group. We also modified the procedure since the exercise will be performed on a mechanical cycle ergometer, facilitating the individualized development for each patient. The work will be overseen by a leading researcher/professional physical educator, following specific medical assessment of the site's nephrology sector.

Marques et al^[18]^ used the Borg perceived effort scale in his program to measure exercise intensity. In our case, the patients will perform aerobic exercise consisting of cyclic movements of the lower limbs. The load will be prescribed by taking into account the Karvonen scale. In Raimundo et al,^[19]^ the subjects were walked on a treadmill for aerobic exercises divided into 5 minutes of warm-up and 25 minutes of exercise, reaching 50% to 70% of their maximal HR (HR_max_). This protocol was modified so that the patients perform the aerobic exercise of cyclical movements of the lower limbs with 45% to 60% intensity of their HR_max_. The total time will be 30 minutes. They will be instructed to remain with arms extended normally in the extension of the chair next to the body, to be accommodated in the best possible way during hemodialysis. The lower limbs will perform the most active part of the exercise by executing a constant rotation of the pedals throughout the aerobic exercise in the mechanical cycler. Patients will be encouraged to increase their intensity according to individual capacity, respecting the principle of biologic individuality. Thus, our protocol consists of developing aerobic exercise 3 times a week, lasting 30 minutes for 3 months, with aerobic intensity, varying from 45% to 60% HR_max_, totaling 36 sessions.

### 2.6 Instruments and data collection

The HRV assessment instrument will be applied at the beginning and end of the aerobic exercise protocol. Initially, the sample size will be 40 adult patients with CRD who undergo hemodialysis in a study with a single screening group. The present study aims to evaluate the effects of supervised aerobic exercise in cycloergometry performed in patients with CRD during hemodialysis sessions, using HRV analysis.

**Figure 1:**
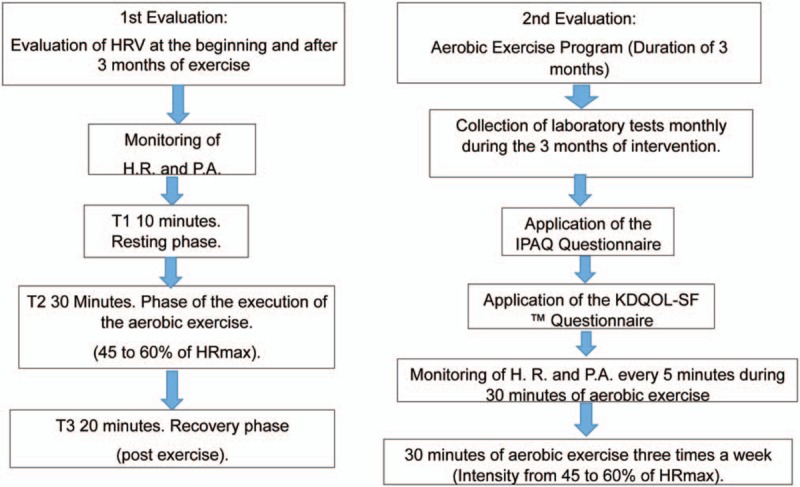
Protocol stages flow chart. HR = heart rate, HR_max_ = maximum heart rate, HRV = heart rate variability, IPAQ = international physical activity questionnaire, KDQOL-SF = kidney disease quality of life short form.

The kidney disease quality of life short form (KDQOL-SF)^[20]^ will specifically evaluate the QoL of the patients with CRD at the Hospital das Clínicas do Rio Branco-Acre. The international physical activity questionnaire (IPAQ) will be used to identify the level of physical activity that the patients are currently experiencing.^[21]^ In addition, information will be collected regarding the laboratory parameters of the patient's charts, and these data will be collected from the month referring to the application of the experimental protocol (Fig. 1).

Regarding the application and evaluation of the exercise, there will be 2 evaluations: The 1st is when the HRV will be determined; the 2nd evaluation will be the development of the exercises during the hemodialysis sessions (Fig. 1).

The 1st evaluation, which consists of capturing the HRV of the exercising patients, will be performed in the first 2 hours of hemodialysis, with a total duration of 1 hour. A cycle ergometer will be used to perform aerobic exercise. All patients will be monitored for cardiovascular function by a portable system (Polar RS 800 CX),^[22]^ which will allow the storage of the data for later analysis of cardiac rhythm variability.

The HR monitoring for subsequent HRV analysis will be obtained by means of a heart rate monitor (RS800CX; Polar Electro Finland Oy, Kempele, Finland) validated to locate the HR for HRV analysis, and collection will be at the beginning of the clinical intervention of the aerobic exercise sessions and after 3 months (end of the intervention), as proposed in this research and shown in Figure 1.

**Figure 2:**
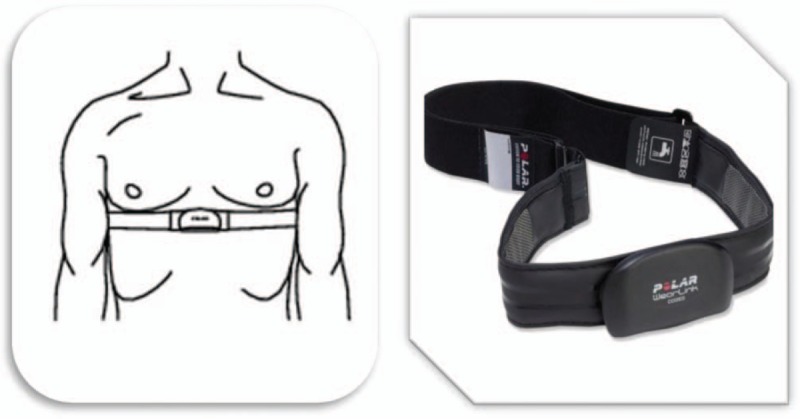
Placement of the strap on the patient. Source: Prepared by the authors, 2018.^[23]^

The cardiofrequency meter will be placed over the patient's praecordial region and secured by an elastic strap to his/her back for proper fixation (Fig. 2).

**Figure 3:**
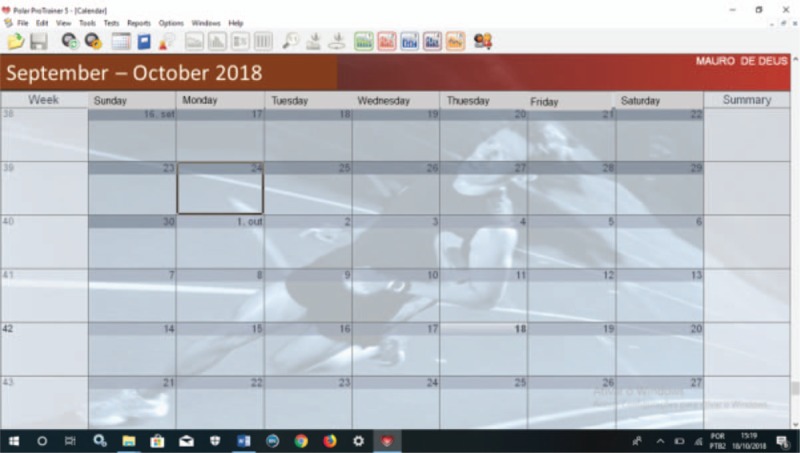
Transfer of data. Source: Prepared by the authors, 2018.

The recorded pulses will be directed to a computer through an infrared signal emitting interface for the analysis of the ventricular function curve (VFC) by the Polar Precision Performance software. For data analysis, the VFC will be filtered at a moderate intensity for the elimination of ectopic beats and/or noises. The HRV analysis will be performed in time and frequency domain and nonlinear methods (Fig. 3).

### 2.7 Protocol's Phase Study

**Figure 4:**
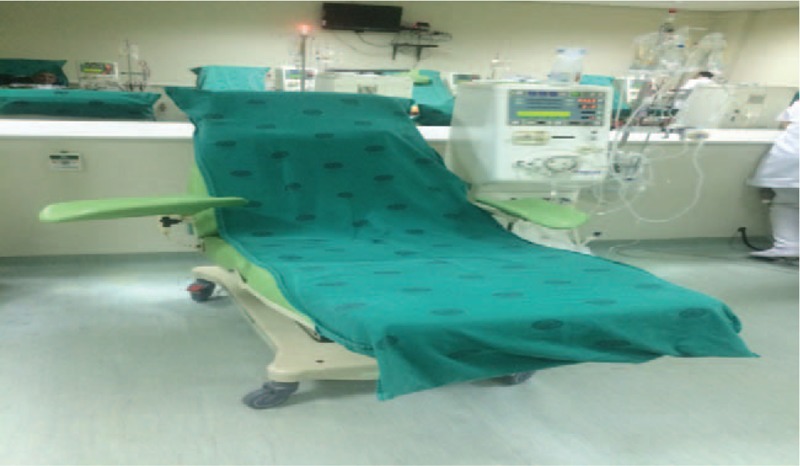
Maca next to the monitoring device where the patient will perform the exercises. Source: Prepared by the authors, 2018.

**Figure 5:**
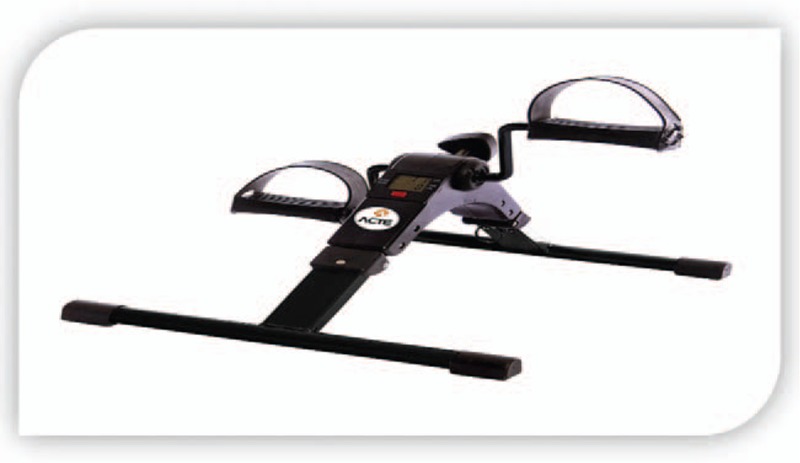
Mini bike compact - E 14. Source: Prepared by the authors, 2018.

This protocol will be done in 3 phases:

- T1: Resting stage (duration of 10 minutes): At this stage, the patient will be instructed to remain as relaxed as possible on the stretcher. They will be instructed to remain with arms extended normally in the extension of the chair next to the body, accommodated in the best possible way.
- T2: Intervention stage (duration of 30 minutes): Patients will perform aerobic exercise consisting of cyclic movements of the lower limbs with 45% to 60% intensity of their HR_max_. They will be instructed to remain with arms extended normally in the extension of the chair next to the body, accommodated in the best way possible (Fig. 4).
- The lower limbs will perform a constant rotation (pedaling) throughout the aerobic exercise in the lower leg cycle ergometer. (Mini Bike Compact - E 14) from ACTE, with multifunction LCD: scan, time, ODO-RPM, distance, calories, speed, measuring (A × C × L) 49 × 41.5 × 34.5 m, to perform aerobic exercise (Fig. 5).
- The patients will start pedaling and will be encouraged to increase the intensity so that it reaches the established zone, and will possibly be able to exceed the upper limit, until the intervention time is over. The load was prescribed by taking into account the Karvonen scale.
- T3: Recovery phase (duration of 20 minutes): Patients will be advised to be as relaxed as possible in the resting phase (recovery). All the patients will be evaluated with the instrument in the initial phase of the research to collect the HRV before the protocol of aerobic exercises proposed during one hour of hemodialysis session (as it appears in the exercise program proposed above). This same evaluation will be carried out with the same characteristics after 3 months.

All patients will be assessed at the beginning of the exercise and every 5 minutes throughout the 30 minutes of the active phase, the HR and BP will be measured by the Bellco Formula Plus dialysis machine (Formula 2000 plus Domus SW 5.8, Mirandola, Italy) (Fig. 4).

At all stages, patients’ complementary data will be collected through their charts as completed by hospital medical staff (body mass, height, weight, age, sex, BP, and HR measurements).

In the 2nd evaluation, the exercise will last 30 minutes. The patients will perform aerobic exercise consisting of cyclical movements of the lower limbs at 45% to 60% intensity of their HR_max_. They will be instructed to remain with arms extended normally in the extension of the chair next to the body, accommodated in the best way possible (Fig. 4). The lower limbs will perform a constant rotation (pedaling) throughout the aerobic exercise in the lower leg cycle ergometer. They will start pedaling and will be encouraged to increase the intensity so that it reaches the established zone, perhaps being able to exceed the upper limit, until the intervention time is over. The load was prescribed by taking into account the Karvonen scale.

All patients will be assessed at the beginning of the exercise and every 5 minutes throughout the 30 minutes of this phase their HR and BP will be measured by the Bellco Formula Plus dialysis machine (Fig. 4).

Patients will undergo 3 weekly sessions of aerobic exercise performed during the first 2 hours of the hemodialysis sessions for 12 weeks (3 months). During collection, all patients will be instructed to avoid intense and continuous conversations.

The IPAQ is recommended by the Clinical Guidelines of the Brazilian Medical Association-Federal Council of Medicine to track the sedentary lifestyle in adults^[24]^ and was proposed by the WHO. The questionnaire will serve to determine the level of physical activity in the population; consisting of sessions with questions related to the time that people spent doing physical activity in a regular week. For this purpose, the short version of the IPAQ^[21]^ will be used.

The KDQOL-SF protocol will be used to evaluate QoL. It is a self-enforcing questionnaire of 80 items divided into 19 dimensions. It includes the short-form health survey as a generic measure and is supplemented with multi-item scales, addressing the particular concerns of chronic kidney patients. The process of coding the questionnaire obeys the “Manual for use and correction of Kidney Disease and Quality of life-Short Form-KDQOL-SF 1.3,” developed by the authors.^[20]^ In general terms, a score is given for each item (or question), and is subsequently transformed into a scale from 0 to 100, in which the value 0 reflects a worse QoL and the value 100 reflects a better QoL. The internal consistency estimated by the KDQOL-SF exceeded 0.80 (range 0.61–0.90).

In addition, information will be collected regarding laboratory parameters of patients’ records, and these data will be collected from the month referring to the application of the experimental protocol.

## 3 Results

### 3.1 Primary outcomes

Recruitment began in November 2017 and is underway. A total of 40 patients should be evaluated and submitted to protocol intervention by the end of this study.

### 3.2 Secondary outcomes

The protocol may provide subsidies for the implementation of new aerobic physical exercise programs for patients with CRD, to help improve their well-being and QoL.

### 3.3 Data analysis

The data will be analyzed using the Statistical Package for Social Science (SPSS), version 22.0. Descriptive analysis will be performed for all variables. Data will be expressed as mean ± standard deviation or median (interquartile range), where appropriate. The distribution of the sample will be tested with the Shapiro–Wilk test. For the comparison of the initial and final values between groups, the unpaired Student *t* test for parametric distributions and the Mann–Whitney test for nonparametric distributions will be applied. Statistical significance will be considered at the *P* < .05 (or 5%) level.

## 4 Discussion

The ANS dysfunction is commonly present in patients with CRD and in patients undergoing hemodialysis and it is an independent factor for a worse prognosis and sudden cardiac death in this population.^[7,8]^ Dysfunction of the ANS has been related to cardiovascular events in patients with CRD.^[7–9]^ Several studies have shown the relationship between HRV reduction and the development of complex arrhythmias in these patients.^[7–9]^ Generally, in the most advanced stage of this pathology, there are changes that are noticed in almost all systems of the body: nervous, cardiovascular, respiratory, musculoskeletal, immunologic, and endocrine/metabolic.^[7–9]^

Physical training in normal individuals leads to increased functional capacity, reduces the risk of cardiovascular diseases, and improves the psychologic structure. Similar benefits have been reported in dialysis patients,^[24]^ even though the actual impact is frequently impaired by the difficulty of adhering to any exercise program, both by sick individuals and by healthy individuals.

Physical activity promotes favorable physiologic adaptations, leading to an improvement in QoL. Following this reasoning, the implementation of an exercise program during hemodialysis sessions is a safe and efficient intervention that helps improve physical performance, nutritional status, QoL, anabolic response, and muscle strength.^[25,26]^

Exercise prescription for patients with CRD is less common than for other chronic diseases; this is notable considering that the levels of physical activity of patients with CRD are significantly lower than among healthy individuals.^[15]^ Despite this, there are indications that exercise can improve physical functioning and positively impact mediators of diseases, comorbidities, and factors associated with the progression of renal disease.^[27]^

Research that can investigate better ways to achieve positive results, with the intention of producing new knowledge and becoming products that will improve the health of the population is important. This could be a new diagnosis, new therapeutic treatments, or actions aimed at promoting the health of the population.^[28,29]^ Corroborating with the authors, the study intends to propose a quality protocol that is both safe and reliable in its execution and data generation, providing reliable information for the health arena. This protocol is expected to be a better indicator of the improvement of the health of patients with CRD who are undergoing hemodialysis treatment.

## Author contributions

**Conceptualization:** Mauro J.D. Morais, Fabiano S. Oliveira, Luiz C. Abreu, Italla M.P. Bezerra, Romeu P.M. Silva, Alliny S. Rodrigues, Rodrigo D. Raimundo, Vitor E. Valenti, Andrés R.P. Riera.

**Data curation:** Mauro J.D. Morais, Fabiano S. Oliveira, Luiz C. Abreu, Italla M.P. Bezerra, Alliny S. Rodrigues, Rodrigo D. Raimundo, Vitor E. Valenti, Andrés R.P. Riera.

**Formal analysis:** Mauro J.D. Morais, Fabiano S. Oliveira, Luiz C. Abreu, Italla M.P. Bezerra, Romeu P.M. Silva, Alliny S. Rodrigues, Rodrigo D. Raimundo, Vitor E. Valenti, Andrés R.P. Riera.

**Funding acquisition:** Mauro J.D. Morais, Fabiano S. Oliveira, Luiz C. Abreu, Italla M.P. Bezerra, Alliny S. Rodrigues, Rodrigo D. Raimundo, Vitor E. Valenti, Andrés R.P. Riera.

**Investigation:** Mauro J.D. Morais, Fabiano S. Oliveira, Luiz C. Abreu, Italla M.P. Bezerra, Alliny S. Rodrigues, Rodrigo D. Raimundo, Vitor E. Valenti, Andrés R.P. Riera.

**Methodology:** Mauro J.D. Morais, Fabiano S. Oliveira, Luiz C. Abreu, Italla M.P. Bezerra, Romeu P.M. Silva, Alliny S. Rodrigues, Rodrigo D. Raimundo, Vitor E. Valenti, Andrés R.P. Riera.

**Project administration:** Mauro J.D. Morais, Fabiano S. Oliveira, Romeu P.M. Silva, Rodrigo D. Raimundo, Vitor E. Valenti, Andrés R.P. Riera.

**Resources:** Mauro J.D. Morais, Fabiano S. Oliveira, Rodrigo D. Raimundo, Andrés R.P. Riera.

**Software:** Mauro J.D. Morais, Fabiano S. Oliveira, Romeu P.M. Silva, Andrés R.P. Riera.

**Supervision:** Mauro J.D. Morais, Fabiano S. Oliveira, Romeu P.M. Silva, Andrés R.P. Riera.

**Validation:** Mauro J.D. Morais, Fabiano S. Oliveira, Andrés R.P. Riera.

**Visualization:** Mauro J.D. Morais, Fabiano S. Oliveira, Andrés R.P. Riera.

**Writing – original draft:** Mauro J.D. Morais, Fabiano S. Oliveira, Andrés R.P. Riera.

**Writing – review & editing:** Mauro J.D. Morais, Fabiano S. Oliveira, Andrés R.P. Riera.
